# Advancing Particle Identification in Cryo-Electron Tomograms with Deep Learning

**DOI:** 10.1063/4.0001096

**Published:** 2025-10-27

**Authors:** Jonathan Schwartz, Saugat Kandel, Hannah Siems, Clinton S Potter, Daniel Serwas, Bridget Carragher, Shawn Zheng, Dari Kimanius

**Affiliations:** Chan Zuckerberg Imaging Institute, Redwood City, CA, USA

## Abstract

Modern cryo-electron microscopy provides the potential to visualize biological macromolecules and protein complexes at near-atomic resolution in their native state [1,2]. Unlike single particle analysis, which requires collecting individual 2D projections of isolated particles, cryo-electron tomography (cryo-ET) paired with sub- tomogram averaging can visualize protein complexes within intact cells at angstrom resolution – given enough copies of the protein of interest are identified [3]. A critical step in this workflow is particle picking, the process of identifying and localizing individual protein complexes within tomograms. Manual particle picking is labor-intensive and classical techniques like template matching often suffer from inaccuracy, long computation times, and reduced performance for smaller complexes. These limitations significantly constrain throughput and scalability, highlighting the demand for more efficient and accurate methods.

To address these challenges, we developed a multi-pronged deep learning approach for organelle segmentation and protein localization. We adapted the Segment Anything Model 2 (SAM2) [4], a foundation model originally developed for video segmentation, to identify lysosomes, vesicles and other subcellular structures with minimal to no additional training in cryo-ET tomograms. While SAM2 excels at broad segmentation tasks across imaging modalities, it is limited in its ability to precisely identify protein complexes, especially in crowded environments or thick samples.

To develop a general methodology for finding protein complexes of varying size, we designed a specialized tool called DeepFindET. This tool employs Bayesian optimization to systematically tailor unique deep- learning model architectures for detecting protein complexes. The resulting 3D convolutional networks integrate physical constraints to sufficiently capture structural information for efficient localization. Further enhancing our methodology, we integrated beneficial training strategies demonstrated by competitors in our Cryo-ET object identification challenge. In particular, the implementation of exponential moving average and advanced data augmentation techniques significantly improved model performance, elevating our score onto the top 10 leaderboard. By employing supervised learning, we guide the models to focus on proteins of interest, enabling targeted insights beyond the capabilities of general-purpose frameworks.

Together, these complementary methods streamline the particle picking process by pairing the versatility of a foundation model with the precision of a specialized model. This combination enables context-aware picking, associating proteins with their corresponding organelles – enhancing both the throughput and contextual accuracy of protein identification. By addressing segmentation and localization as distinct but interconnected tasks, our approach underscores the symbiotic relationship between foundation and specialized models, demonstrating how their integration can advance particle picking workflows, and provide deeper insights into cellular architectures.

## References

[c1] Mahamid, J., et.al. *Science* 351, 969 (2016). 26917770 10.1126/science.aad8857

[c2] Xue, L., et. al. *Nature* 610, 205 (2022). 36171285 10.1038/s41586-022-05255-2 PMC9534751

[c3] Zivanov, J. et. al. eLife 11, e83724 (2022). 10.7554/eLife.83724 36468689 PMC9815803

[c4] Ravi, N. et. al. arXiv (2024).

